# The ubiquitin isopeptidase USP10 deubiquitinates LC3B to increase LC3B levels and autophagic activity

**DOI:** 10.1016/j.jbc.2021.100405

**Published:** 2021-02-10

**Authors:** Rui Jia, Juan S. Bonifacino

**Affiliations:** Neurosciences and Cellular and Structural Biology Division, Eunice Kennedy Shriver National Institute of Child Health and Human Development, National Institutes of Health, Bethesda, Maryland, USA

**Keywords:** USP10, LC3, ubiquitin, deubiquitination, autophagy, protein aggregation, CRISPR/cas, ALIS, aggresome-like induced structures, ATG, autophagy-related gene, CHX, cycloheximide, DUB, deubiquitinating enzyme, KD, knockdown, KO, knockout, LC3B, microtubule-associated protein 1 light chain 3 beta or MAP1LC3B, MAGeCK, model-based analysis of genome-wide CRISPR-Cas9 knockout, NGS, Next-generation sequencing, RRA, robust ranking aggregation, tfLC3B, tandem fluorescent-tagged LC3B, USP10, ubiquitin-specific peptidase 10

## Abstract

Components of the autophagy machinery are subject to regulation by various posttranslational modifications. Previous studies showed that monoubiquitination of LC3B catalyzed by the ubiquitin-activating enzyme UBA6 and ubiquitin-conjugating enzyme/ubiquitin ligase BIRC6 targets LC3B for proteasomal degradation, thus reducing LC3B levels and autophagic activity under conditions of stress. However, mechanisms capable of counteracting this process are not known. Herein, we report that LC3B ubiquitination is reversed by the action of the deubiquitinating enzyme USP10. We identified USP10 in a CRISPR-Cas9 knockout screen for ubiquitination-related genes that regulate LC3B levels. Biochemical analyses showed that silencing of USP10 reduces the levels of both the LC3B-I and LC3B-II forms of LC3B through increased ubiquitination and proteasomal degradation. In turn, the reduced LC3B levels result in slower degradation of the autophagy receptors SQSTM1 and NBR1 and an increased accumulation of puromycin-induced aggresome-like structures. Taken together, these findings indicate that the levels of LC3B and autophagic activity are controlled through cycles of LC3B ubiquitination and deubiquitination.

Autophagy is a cellular process for the lysosomal degradation of cytoplasmic materials (*i.e.*, “cargos”) such as organelles, protein aggregates, and intracellular pathogens (1, 2). This process involves engulfment of the cargos into double-membraned vesicles named autophagosomes. The autophagosomes subsequently fuse with lysosomes to form autolysosomes, where the cargos are degraded and the products of degradation recycled (1, 2). Autophagy is critical for cellular homeostasis, and autophagic defects underlie the pathogenesis of many human diseases, including neurodegenerative disorders, cardiomyopathy, cancer, type-II diabetes, and immune system disorders (3).

The mechanism of autophagy is mediated by a core machinery comprising over 30 different proteins (1, 2). In addition, many other proteins act as regulators by altering the activities of core machinery components. Among these regulators are enzymes that catalyze posttranslational modifications such as phosphorylation/dephosphorylation, acetylation/deacetylation, ubiquitination/deubiquitination, lipidation/delipidation, and proteolysis (4, 5).

A key target of regulatory modifications is the autophagy protein LC3B (abbreviation for microtubule associated protein 1 light chain 3 beta or MAP1LC3B), the best studied of six human orthologs of yeast Atg8, the others being LC3A, LC3C, GABARAP, GABARAPL1, and GABARAPL2 (1, 2). LC3B participates in cargo engulfment, autophagosome maturation, and autophagosome–lysosome fusion (1, 2). Upon induction of autophagy, LC3B is converted from a cytosolic LC3B-I form to a membrane-bound LC3B-II form, which is the active species in autophagy (1, 2), LC3B is phosphorylated at different amino-acid residues, in some cases with demonstrated consequences on autophagy. For example, phosphorylation of LC3B on Thr-50 by STK3 and STK4 (serine/threonine kinase 3 and 4), PKCζ (protein kinase Cζ), or NEK9 (NIMA-related kinase 9) regulates autophagosome–lysosome fusion and binding of LC3B to several LC3-interacting region (LIR)-containing proteins, such as SQSTM1 (sequestosome-1, also known as p62), NBR1 (neighbor of BRCA1 gene), FYCO1 (FYVE and coiled-coil domain-containing 1), and ATG4 (autophagy gene 4 product) (6). LC3B is also phosphorylated on Thr-6 and Thr-29 by PKC (protein kinase C), but these modifications do not seem to affect autophagy (7).

Acetylation of LC3B also participates in autophagy regulation. Acetyl groups are covalently linked to LC3B on Lys-49 and Lys-51 by EP300 (E1A binding protein p300) and CREBBP (CREB binding protein) acetylases (8, 9) and removed from LC3B by the SIRT1 (NAD-dependent sirtuin 1) deacetylase (10). Acetylated LC3B mainly accumulates in the nucleus in an inactive form. Deacetylation of LC3B promotes its redistribution to the cytoplasm, where it participates in autophagy (9). Accordingly, EP300 knockdown (KD) stimulates starvation-induced autophagy (8), whereas SIRT1 KD results in autophagy defects (10).

Recently, autophagy was also shown to be regulated by ubiquitination. We and others found that LC3B is monoubiquitinated by the concerted action of the UBA6 E1 ubiquitin (Ub)-activating enzyme and the BIRC6 hybrid E2 ubiquitin-conjugating enzyme/E3 Ub ligase (11, 12), and polyubiquitinated by the von Hippel–Lindau (VHL) tumor suppressor E3 Ub ligase (13). Both monoubiquitination and polyubiquitination lead to proteasomal degradation of LC3B, with consequent decrease in autophagic activity (11, 12, 13). In general, protein ubiquitination is reversed by Ub removal catalyzed by a family of isopeptidases known as deubiquitinating enzymes (DUBs) (14). However, this has not yet been shown to be the case for ubiquitinated LC3B. Herein, we report the results of a CRISPR-Cas9 KO screen that identifies USP10 as a DUB that deubiquitinates LC3B, thus increasing LC3B levels and autophagic activity.

## Results and discussion

**Figure 1:**
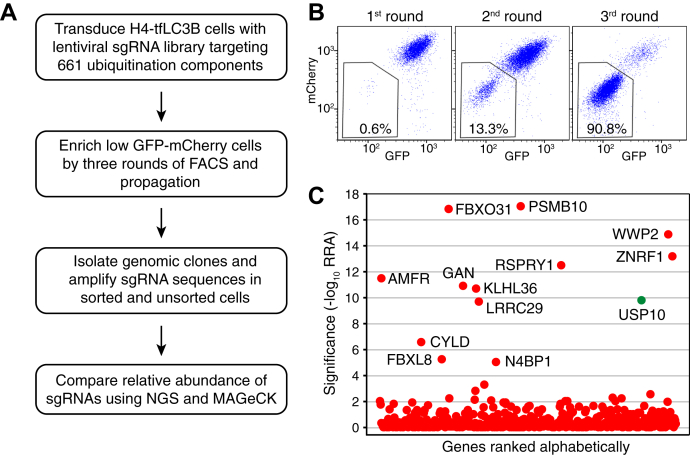
**CRISPR-Cas9 KO screen for ubiquitination-related proteins that regulate LC3B levels.***A*, workflow of CRISPR-Cas9 screen with a ubiquitination sgRNA library. For details of the screen, see Experimental procedures. *B*, FACS profiles showing the enrichment of a population of H4-tfLC3B cells with low levels of GFP and mCherry signals from 0.6% to 90.8% after three rounds of selection and propagation. *C*, ranking of genes from the CRISPR-Cas9 screen based on the Robust Ranking Aggregation (RRA) score calculated using the MAGeCK algorithm. The deubiquitinating enzyme USP10 is indicated with a *green dot*. See Tables S1 and S2 for additional information on the top hits.

To identify potential LC3B DUBs, we conducted a CRISPR-Cas9 KO screen using a human H4 neuroglioma cell line that expresses LC3B endogenously tagged with tandem GFP-mCherry (H4-tfLC3B cells) (12). These cells were mutagenized with a lentiviral CRISPR-Cas9 KO library targeting 661 ubiquitination-related genes (12) (Fig. 1*A*). The screen was based on the hypothesis that depletion of specific DUBs would increase LC3B ubiquitination and degradation, thus decreasing GFP and mCherry fluorescence signals. Mutagenized H4-tfLC3B cells exhibiting low GFP-mCherry fluorescence were collected by fluorescence-activated cell sorting (FACS). These cells were then propagated and subjected to two more rounds of sorting and propagation, until the population of low GFP-mCherry cells was enriched to 90.8% (Fig. 1, *A* and *B*). The abundance of every single-guide RNA (sgRNA) in sorted cells relative to unsorted cells was determined by next-generation sequencing (NGS) and the MAGeCK (Model-based Analysis of Genome-wide CRISPR-Cas9 Knockout) algorithm (15) (Fig. 1*C*, Tables S1 and S2). The top-scoring DUB in this screen (ranked number 9 in order of significance) was USP10. The other proteins among the top ten hits were the immunoproteasome subunit beta type-10 (PSMB10) and components of several E3 ubiquitin ligases (Table S2); because the aim of the present study was to identify a putative LC3B-deubiquitinating enzyme, the functional significance of these other proteins was not investigated.

**Figure 2:**
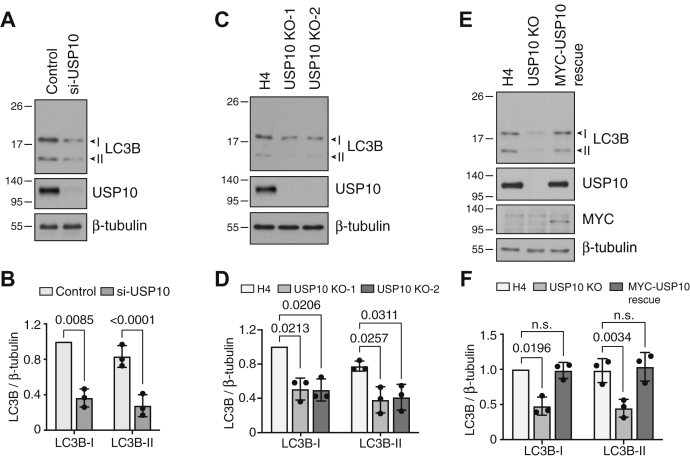
**Silencing USP10 reduces the levels of both LC3B-I and LC3B-II.***A*, H4 cells were transfected with control or USP10 SMARTpool siRNAs. After 48 h, cells were analyzed by SDS-PAGE and immunoblotting with antibodies to the indicated proteins. Positions of the I and II forms of LC3B are shown with *arrowheads*. *B*, quantification of the ratio of LC3B-I and LC3B-II to β-tubulin. The LC3B-I to β-tubulin ratio for control siRNA transfection was arbitrarily set at 1. Bars represent the mean ± SD from three independent experiments such as that shown in *A*. Individual values from each experiment are represented by *dots*. The indicated *p*-value relative to LC3B-I in the control was calculated using a one-sample *t*-test; the *p*-value relative to LC3B-II in the control was calculated using an unpaired Student’s *t*-test. *C*, SDS-PAGE and immunoblot analysis of WT H4 cells and two clones of USP10-KO H4 cells using antibodies to the indicated proteins. *D*, quantification of the ratio of LC3B-I and LC3B-II to β-tubulin from three independent experiments such as that shown in *C*. Calculations and statistics were done as described in *B*. *E*, WT, USP10-KO, and MYC-USP10-rescued USP10-KO H4 cells were analyzed by SDS-PAGE and immunoblotting with antibodies to the indicated proteins. *F*, quantification of the ratio of LC3B-I and LC3B-II to β-tubulin from three independent experiments such as that shown in *E*. Calculations and statistics were done as described in *B*. In *A*, *C*, and *E*, the positions of molecular mass markers (in kDa) are indicated on the left.

To confirm a role of USP10 in the regulation of LC3B levels, we silenced USP10 expression by transfecting H4 cells with USP10 siRNA. We observed that USP10 silencing decreased the endogenous levels of both the cytosolic LC3B-I and membrane-bound LC3B-II forms by ∼2–2.5-fold (Fig. 2, *A* and *B*). Similar decreases in LC3B-I and LC3B-II levels were observed upon silencing of USP10 in the HeLa human cervical carcinoma and HEK293T human embryonic kidney cell lines (Fig. S1, *A–C*). Furthermore, CRISPR-Cas9 KO of USP10 in H4 cells resulted in approximately twofold decreases in both LC3B-I and LC3B-II endogenous levels (Fig. 2, *C* and *D*). Stable expression of MYC-USP10 in USP10-KO cells restored levels of LC3B-I and LC3B-II to those in WT cells (Fig. 2, *E* and *F*). Analyses of other Atg8-family members showed that USP10 KO in H4 cells also reduced the levels of LC3A, but not GABARAP and GABARAPL1 (Fig. S2, *A–E*). The effect of USP10 KO on the levels of LC3C and GABARAPL2 could not be examined because of lack of suitable antibodies for immunoblotting. Finally, we observed that transfection of USP10-KO H4 cells with siRNA to UBA6, the Ub-activating enzyme that participates in LC3B ubiquitination (12), increased the levels of both LC3B-I and LC3B-II (Fig. S3, *A* and *B*). These results thus demonstrated that USP10 depletion reduced the levels of endogenous LC3B, an effect that was opposite to that caused by depletion of the ubiquitinating enzymes UBA6 or BIRC6 (11, 12).

**Figure 3:**
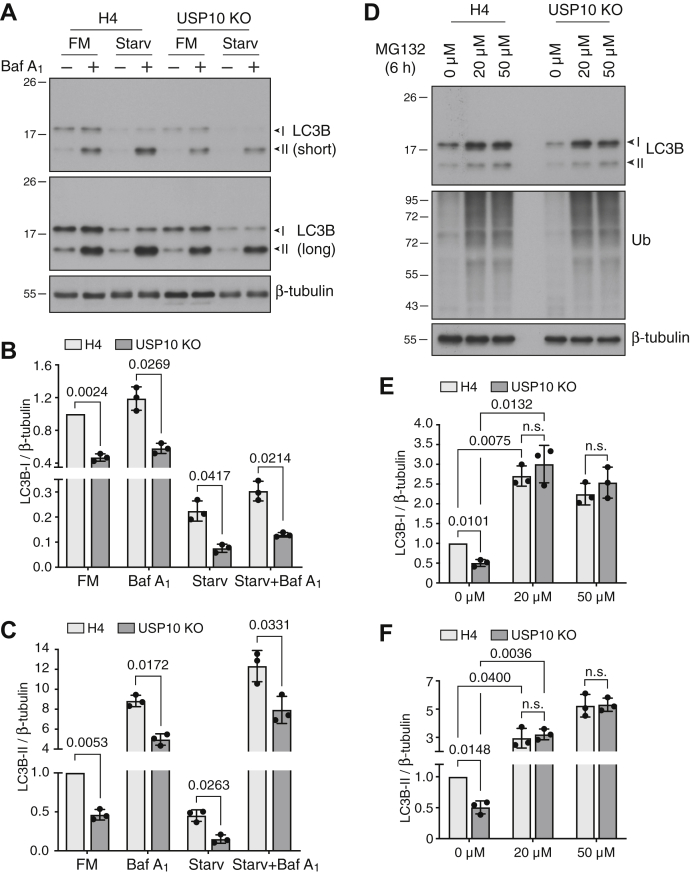
**Reduced LC3B-II levels in USP10-KO cells under conditions of bafilomycin A**_**1**_**treatment and starvation.***A*, WT and USP10-KO H4 cells were incubated with 50 nM bafilomycin A_1_ (Baf A_1_), starvation medium (Starv), or a combination of both for 2 h. Cells were then analyzed by SDS-PAGE and immunoblotting with antibodies to LC3B and β-tubulin. FM stands for fed medium (complete medium). *B* and *C*, quantification of the ratio of LC3B-I (*B*) and LC3B-II (*C*) to β-tubulin. The ratio for WT H4 cells in FM was arbitrarily set at 1. Bars represent the mean ± SD from three independent experiments such as that shown in *A*. Individual values from each experiment are represented by *dots*. The indicated *p*-value relative to WT H4 cells in the FM condition was calculated using a one-sample *t*-test; other *p*-values were calculated using an unpaired Student’s *t*-test. *D*, WT and USP10-KO H4 cells were incubated with 0, 20, or 50 μM MG132 for 6 h and analyzed by SDS-PAGE and immunoblotting with antibodies to the indicated proteins. The accumulation of polyubiquitinated proteins (*middle panel*) was examined as a control for MG132 activity. *E* and *F*, quantification of the ratio of LC3B-I (*E*) and LC3B-II (*F*) to β-tubulin. The ratio for WT H4 cells without MG132 was arbitrarily set at 1. Bars represent the mean ± SD from three independent experiments such as that shown in *D*. Individual values from each experiment are represented by *dots*. The indicated *p*-values relative to WT H4 cells in the absence of MG132 were calculated using a one-sample *t*-test; other *p*-values were calculated using an unpaired Student’s *t*-test. In *A* and *D*, the positions of molecular mass markers (in kDa) are indicated on the left.

LC3B can be turned over by both lysosomal (16) and proteasomal degradation (11, 12, 13). To examine which pathway was responsible for the reduced levels of LC3B in USP10-deficient cells, we tested the effect of specific inhibitors. LC3B-I is converted to LC3B-II during autophagosome formation, and LC3B-II is eventually degraded, together with autophagy receptors and substrates, upon fusion of autophagosomes with lysosomes (16, 17). Inhibition of lysosomal degradation by treatment with the v-ATPase inhibitor bafilomycin A1 dramatically increased the levels of LC3B-II in both WT and USP10-KO H4 cells, indicative of the accumulation of undegraded LC3B-II in lysosomes in both cell lines (*i.e.*, a measure of “autophagic flux”) (Fig. 3, *A–C*). Importantly, the levels of LC3B-II under these conditions were still lower in USP10-KO cells than in WT cells (Fig. 3, *A–C*). Furthermore, autophagy induction by removal of amino acids and serum from the medium (*i.e.*, “starvation”) decreased the levels of LC3B-II in both WT and USP10-KO cells due to increased LC3B consumption (Fig. 3, *A–C*); however, the levels of LC3B-II remained lower in USP10-KO relative to WT cells (Fig. 3, *A–C*). Finally, combined treatment with bafilomycin A_1_ and nutrient starvation resulted in greater accumulation of LC3B-II in both WT and USP10-KO cells, but, again, LC3B-II levels were lower in USP10-KO than in WT cells (Fig. 3, *A–C*). These findings indicated that the decreased levels of LC3B in USP10-deficient cells were not due to increased lysosomal/autophagic degradation, but rather preceded the engagement of LC3B in autophagy.

To determine if the reduced levels of LC3B in USP10-KO cells were due to increased proteasomal degradation, we incubated WT and USP10-KO cells for 6 h with 20 or 50 μM of the proteasome inhibitor MG132. We observed that treatment with either concentration of MG132 increased the amounts of LC3B-I and LC3B-II to similar levels in both WT and USP10-KO cells (Fig. 3, *D–F*), indicating that differences in LC3B levels between untreated WT and USP10-KO cells were due to proteasomal degradation. It is worth noting that MG132-treated cells accumulated unconjugated LC3B instead of monoubiquitinated LC3B (Fig. 3*D*), a fact that could be explained by removal of the Ub moiety by proteasomal DUBs (18).

For further proof that the increase in LC3B levels caused by treatment with MG132 was not due to inhibition of autophagy, we examined the effect of knocking out ATG7, an E1-like enzyme that participates in lipidation (*i.e.*, conversion of LC3B-I to LC3B-II) and eventual autophagic degradation of LC3B (1, 2). We observed that incubation with 10 μM MG132 caused similar fold increases in LC3B-I levels in both WT and ATG7-KO cells (Fig. S4, *A–C*), confirming that the effect of MG132 was independent of autophagy.

**Figure 4:**
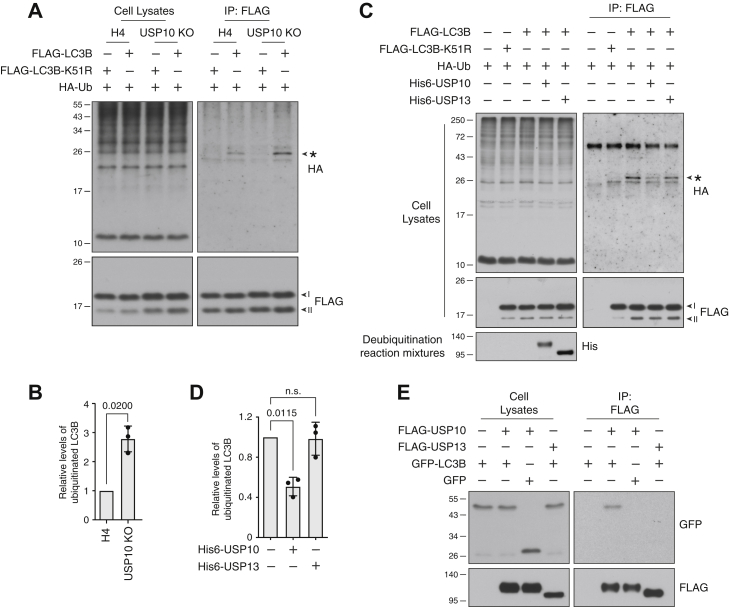
**USP10 deubiquitinates LC3B.***A*, WT and USP10-KO H4 cells were transfected with plasmids encoding FLAG-LC3B or FLAG-LC3B-K51R, and HA-Ub. After 24 h, cells were incubated with 5 μM MG132 for 18 h. Cell lysates were immunoprecipitated with antibody to the FLAG epitope. Cell lysates and immunoprecipitates were analyzed by SDS-PAGE and immunoblotting with antibodies to FLAG and HA epitopes. The *asterisk* indicates the specific ubiquitinated LC3B band. Notice that USP10 KO increases the levels of ubiquitinated LC3B, but that under conditions of LC3B overexpression, it does not decrease total LC3B levels, probably because LC3B overexpression overwhelms the regulatory effect of ubiquitination. *B*, quantification of levels of ubiquitinated LC3B (immunoblotting with anti-HA antibody) normalized to nonubiquitinated LC3B (immunoblotting with anti-FLAG antibody). The relative level in WT H4 cells was arbitrarily set at 1. Bars represent the mean ± SD from three independent experiments such as that shown in *A*. Individual values from each experiment are represented by *dots*. The indicated *p*-value was calculated using a one-sample *t*-test. *C*, WT H4 cells were transfected with plasmids encoding FLAG-LC3B and FLAG-LC3B-K51R, and HA-Ub. Cell lysates were analyzed by immunoprecipitation with antibody to the FLAG epitope. The FLAG-LC3B bound to beads was incubated with recombinant His6-USP10 or His6-USP13, and the deubiquitination reaction mixtures were collected for further analysis. FLAG-LC3B was eluted from beads with 3xFLAG peptide. Cell lysates, elution products, and deubiquitination reaction mixtures were analyzed by SDS-PAGE and immunoblotting with antibodies to the FLAG, HA, and His6 epitopes. The *asterisk* indicates ubiquitinated LC3B. *D*, quantification of relative levels of ubiquitinated LC3B normalized to nonubiquitinated LC3B. The relative level for control without deubiquitinating enzymes was arbitrarily set at 1. Bars represent the mean ± SD from three independent experiments such as that shown in *C*. Individual values from each experiment are represented by *dots*. The indicated *p*-values were calculated using a one-sample *t*-test. *E*, WT H4 cells were transfected with plasmids encoding FLAG-USP10, FLAG-USP13, GFP-LC3B, and GFP. Cell lysates were immunoprecipitated with antibody to the FLAG epitope. Cell lysates and immunoprecipitates were analyzed by SDS-PAGE and immunoblotting with antibodies to GFP and FLAG epitope. In *A*, *C*, and *E*, the positions of molecular mass markers (in kDa) are indicated on the left.

Next, we examined if USP10 altered the ubiquitination state of LC3B. To this end, we cotransfected WT or USP10-KO cells with plasmids encoding HA-tagged Ub (HA-Ub) and either FLAG-tagged LC3B (FLAG-LC3B) or FLAG-LC3B having a mutation in the ubiquitin-acceptor Lys-51 to Arg (FLAG-LC3B-K51R) (12). Cells were incubated with MG132 prior to harvesting. Cell extracts were subjected to immunoprecipitation with antibody to the FLAG epitope followed by immunoblotting with antibody to the HA epitope. We observed that FLAG-LC3B, but not FLAG-LC3B-K51R, was modified with HA-Ub in WT cells (Fig. 4, *A* and *B*), as previously reported (12). Importantly, FLAG-LC3B ubiquitination was increased in USP10-KO cells (Fig. 4, *A* and *B*). In addition, we found that ubiquitination of FLAG-LC3A and FLAG-LC3C was also increased in USP10-KO cells (Fig. S5). GABARAP, GABARAPL1, and GABARAPL2 are not ubiquitinated (12), and were therefore not included in these analyses. These results thus indicated that USP10 reduces the ubiquitination of not only LC3B, but also LC3A and LC3C.

We also used an *in vitro* assay to test the effect of treating HA-Ub-conjugated FLAG-LC3B with recombinant His6-tagged USP10 (His6-USP10) or His6-tagged USP13 (His6-USP13) (specificity control). We observed that addition of His6-USP10, but not His6-USP13, reduced the amount of HA-Ub-conjugated FLAG-LC3B (Fig. 4, *C* and *D*), consistent with USP10 specifically catalyzing the deubiquitination of LC3B. Finally, we found that GFP-tagged LC3B (GFP-LC3B) co-immunoprecipitated with FLAG-USP10 but not FLAG-USP13 (Fig. 4*E*). Taken together, the above experiments demonstrated that USP10 specifically deubiquitinates LC3B both *in vivo* and *in vitro*.

**Figure 5:**
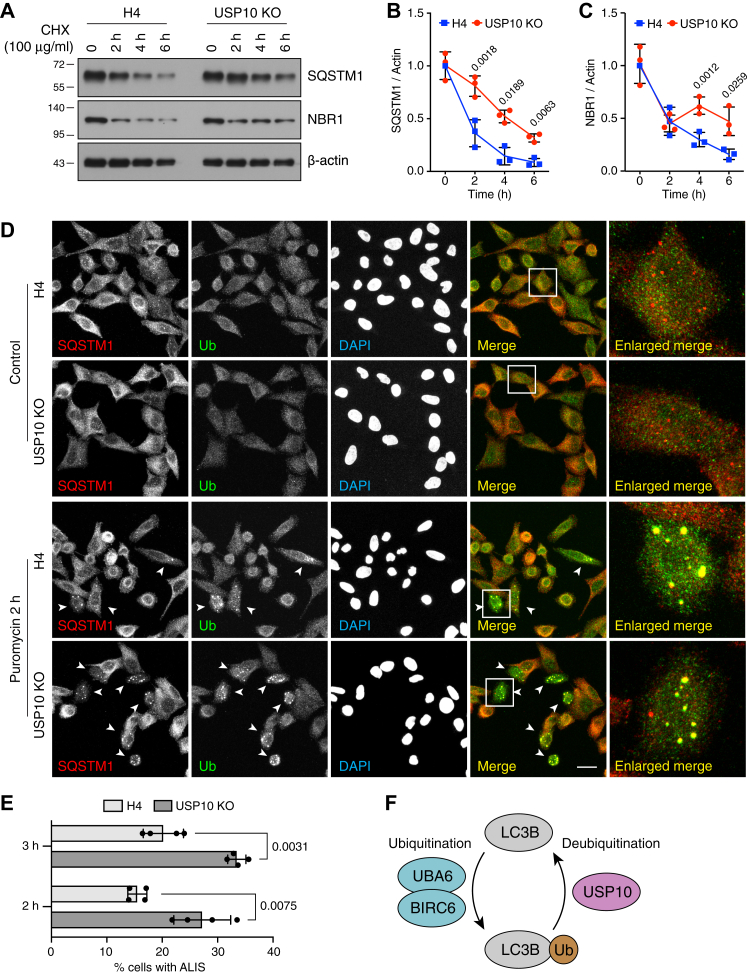
**USP10 KO increases the accumulation of autophagy receptors and puromycin-induced aggregates.***A*, WT and USP10-KO H4 cells were incubated with 100 μg/ml cycloheximide (CHX) for 0, 2, 4, or 6 h and analyzed by SDS-PAGE and immunoblotting for SQSTM1, NBR1 and β-actin. The positions of molecular mass markers (in kDa) are indicated on the left. *B* and *C*, quantification of the ratio of SQSTM1 (*B*) and NBR1 (*C*) to β-actin at different time points. Values for WT H4 cells at time 0 were arbitrarily set to 1 and represent the mean ± SD from three independent experiments such as that shown in *A*. Individual values from each experiment are represented by *dots*. The indicated *p*-values were calculated using an unpaired Student’s *t*-test. *D*, confocal microscopy of WT and USP10-KO H4 cells incubated for 2 h without (control) or with 5 μg/ml puromycin, prior to immunostaining for SQSTM1 and Ub. Scale bar: 20 μm. Cells containing aggresome-like induced structures (ALIS) are indicated with *arrowheads*. The right-most column shows fivefold enlargements of the boxed areas. *E*, quantification of the percentage of ALIS-containing WT and USP10-KO H4 cells treated with 5 μg/ml puromycin for 2 or 3 h from experiments such as that shown in *D*. Bars represent the mean ± SD of the percentages of cells with ALIS from four independent experiments counting more than 1200 cells. Individual values from each experiment are represented by *dots*. The indicated *p*-values were calculated using an unpaired Student’s *t*-test. *F*, schematic representation of the LC3B ubiquitination/deubiquitination cycle driven by UBA6-BIRC6 and USP10.

The degradation of selected cargos by autophagy is achieved through recognition by various autophagy receptors, which are eventually degraded in autolysosomes together with their cargos and with LC3B (1, 2). To investigate the effect of USP10 KO on autophagy-receptor degradation, we incubated WT and USP10-KO H4 cells with the translation inhibitor cycloheximide (CHX) for 0, 2, 4, and 6 h in complete medium (Fig. 5, *A-C*) or starvation medium (Fig. S6, *A–C*) and determined the endogenous levels of the autophagy receptors SQSTM1 and NBR1 by immunoblotting (Figs. 5, *A–C* and S6, *A–C*). We observed that, in complete medium, the steady-state levels of SQSTM1 and NBR1 at time 0 were similar in WT and USP10-KO cells, suggesting that the lower level of LC3B in USP10-KO cells was sufficient to maintain basal autophagic activity. Treatment with cycloheximide caused a time-dependent decrease in the levels of SQSTM1 and NBR1 in both WT and USP10-KO cells, but the decrease was slower in USP10-KO cells (Fig. 5, *A-C*). Similar observations were made by CHX treatment in starvation medium (Fig. S6, *A–C*). These experiments indicated that the lower levels of LC3B in USP10-KO cells limit the availability of LC3B for recruitment of SQSTM1 and NBR1 to autophagosomes and their eventual degradation in autolysosomes under conditions of protein synthesis inhibition.

Aggresome-like induced structures (ALIS) are protein aggregates that can be induced under conditions of stress, such as premature translation termination by treatment with puromycin (19). Puromycin-induced ALIS are usually modified by ubiquitination, selectively recognized by autophagy receptors such as SQSTM1 and NBR1, and subsequently degraded by autophagy (19, 20). To examine the effect of USP10 KO on the accumulation of ALIS, we incubated WT and USP10-KO H4 cells with 5 μg/ml puromycin for 2 and 3 h and visualized the distribution of endogenous Ub and SQSTM1 by immunofluorescence microscopy. We observed that, under control conditions, neither WT nor USP10-KO H4 cells showed any ALIS (Fig. 5*D*). After 2 h of incubation with puromycin, however, 15.5% of WT cells and 27.2% in USP10-KO cells showed ALIS accumulation (Fig. 5, *D* and *E*). These percentages increased to 20.2% of WT and 33.5% of USP10-KO cells, respectively, after 3 h puromycin incubation (Fig. 5, *D* and *E*). These observations indicated that USP10 KO increased ALIS accumulation, consistent with an impaired autophagic response to the formation of abnormal protein aggregates.

Taken together, the results presented here indicate that USP10 is required for maintenance of higher steady-state levels of LC3B by deubiquitinating LC3B and reducing its targeting for proteasomal degradation. Higher LC3B levels enable increased autophagy flux under both basal and starvation conditions. This ability is not necessary for maintaining normal levels of the autophagy receptors SQSTM1 and NBR1 and for clearance of protein aggregates in untreated cells, but becomes important for these functions upon inhibition of translation by treatment with cycloheximide or puromycin. Hence, USP10 enables an enhanced autophagic response under stress conditions. This function of USP10 counters that of UBA6, BIRC6, and VHL, which mediate ubiquitination and proteasomal degradation of LC3B, thus decreasing stress-induced autophagy (11, 12, 13) (Fig. 5*F*). Although most of our studies were done on LC3B, we also showed that USP10 (this study), UBA6, and BIRC6 (12) control the ubiquitination and/or levels of LC3A and LC3C, suggesting that the same regulatory mechanism acts on the entire LC3 subset of the Atg8 family. In contrast, the GABARAP subset is not subject to this type of regulation.

USP10 has also been shown to enhance autophagy by deubiquitinating beclin-1, a component of class-III PI3K complexes that catalyze the formation of phosphatidylinositol 3-phosphate at both autophagy initiation and autophagosome maturation steps (21). Moreover, beclin-1 and USP10 are involved in a potential feedforward mechanism in which beclin-1 stabilizes USP13, which in turn deubiquitinates and stabilizes USP10, leading to increased beclin-1 levels and activity (21). Finally, USP10 deubiquitinates and activates the autophagy-promoting kinase AMPK (22). Thus, USP10 exerts broad proautophagic effects by deubiquitinating, stabilizing, and/or activating various components of the autophagy machinery.

Our results are consistent with those of previous studies that showed that the expression levels of LC3 proteins influence autophagy activity. For example, KD of LC3 proteins caused elevation of SQSTM1 levels and accumulation of polyubiquitinated aggregates (23, 24, 25). Conversely, overexpression of LC3 proteins enhanced autophagic activity, increasing the clearance of pathogenic aggregates and reducing inflammation and tissue injury (26). In general, drugs that enhance autophagy have been proposed as potential agents for the treatment of various diseases; however, most of them have side effects that make them unsuitable for clinical use (27, 28). Therefore, there is interest in identifying novel targets for pharmacologic manipulation of autophagy. We think that small molecules that increase LC3 levels through activation of USP10 or inhibition of UBA6, BIRC6, and VHL could be candidates for a novel class of autophagy enhancers. The fact that USP10 also positively regulates beclin-1 and AMPK (21, 22) would make pharmacologic activators of USP10 particularly effective as autophagy enhancers.

## Experimental procedures

### Cell culture and transfection

Cell culture and transfection were performed as previously described (12). H4 (ATCC, HTB-148), HeLa (ATCC, CCL-2), HEK293 T (ATCC, CRL-11268), H4-tfLC3B (12), USP10-KO H4 (this study), and ATG7-KO HeLa (kind gift of Elodie Mailler, NICHD, NIH) cells were grown in Dulbecco's modified Eagle's medium (DMEM, Corning, 15-013-CV) containing 10 % fetal bovine serum (FBS, Corning, 35-011-CV), 100 IU/ml penicillin, 100 μg/ml streptomycin (Corning, 30-002-CI), and 2 mM L-glutamine (Corning, 25-005-CI) at 37 °C, 5 % CO_2_. Transfection of USP10 siRNA (Horizon, L-006062-00-0005), UBA6 siRNA (Thermo Fisher Scientific, 4392420-s30516), and nontargeting siRNA (UGGUUUACAUGUCGACUAAUUU, Eurofins Scientific) was performed with Oligofectamine (Thermo Fisher Scientific, 12252011) according to the manufacturer’s instructions. Starvation was performed by incubating cells with DMEM without amino acids and serum (MyBioSource, MBS653087).

### Antibodies, chemicals, and recombinant proteins

We used primary antibodies to the following proteins: LC3A (Cell Signaling Technology, 4599), LC3B (Cell Signaling Technology, 3868), GABARAP (Cell Signaling Technology, 13733), GABARAPL1 (Cell Signaling Technology, 26632), UBA6 (Cell Signaling Technology, 13386), ATG7 (Cell Signaling Technology, 8558), USP10 (Cell Signaling Technology, 8501), β-tubulin (Cell Signaling Technology, 2146), MYC epitope (Santa Cruz Biotechnology, sc-789), Ub (for immunoblotting) (Thermo Fisher Scientific, 13-1600), Ub (for immunofluorescence) (Enzo Life Sciences, BML-PW8805-0500), HA epitope (BioLegend, 901501), FLAG epitope (Sigma-Aldrich, F1804), His-Tag (Cell Signaling Technology, 2365), GFP (Thermo Fisher Scientific, A11122), SQSTM1 (for immunoblotting) (BD Biosciences, 610833), SQSTM1 (for immunofluorescence) (Enzo Life Sciences, BML-PW9860-0100), NBR1 (Cell Signaling Technology, 9891), β-actin (Cell Signaling Technology, 3700). HRP-conjugated anti-mouse IgG (NEF822001EA) and anti-rabbit IgG (NEF812001EA) secondary antibodies were purchased from PerkinElmer. Alexa Fluor 555-conjugated goat anti-rabbit IgG (A-21428) and Alexa Fluor 488 conjugated goat anti-mouse IgG (A-11001) secondary antibodies were purchased from Thermo Fisher Scientific.

Bafilomycin A1 (B1793), MG132 (M7449), cycloheximide (C4859), puromycin (P8833), hexadimethrine bromide (Polybrene) (H9268), and fibronectin (F2006) were purchased from Sigma-Aldrich. His6-USP10 (E-592) and His6-USP13 (E-588) recombinant proteins were purchased from Boston Biochem.

### Plasmids

Plasmids encoding FLAG-HA-USP10 (22543; from Wade Harper), FLAG-HA-USP13 (22568; from Wade Harper), pSpCas9 (BB)-2A-GFP (40823; from Noboru Mizushima), pMD2.G (12259; from Didier Trono), psPAX (12260; from Didier Trono), HA-Ub (18712; from Edward Yeh) were obtained from Addgene. Plasmids encoding GFP-LC3B (29), FLAG-LC3B (12) and FLAG-LC3B-K51R (12) were described previously.

pQCXIP-MYC-USP10 and pQCXIP-FLAG-USP10 were generated by subcloning coding sequences for human USP10 from FLAG-HA-USP10 into pQCXIP vector (TaKaRa, S3145) with an N-terminal MYC tag or FLAG tag. The coding sequence of USP10 with MYC tag was generated by PCR with primers CGGATCCTATCTCGAGATTAGAGATCCTCCTCTGAGATGAGTTTTTGTTCGGATCCGCTTCCCAGCAGGTCCACTCGGCG and CGGATCCTATCTCGAGATTACAGCAGGTCCACTCGGCG, while the coding sequence of USP10 with FLAG tag was generated by PCR with primers GCAGGAATTGATCCGCATGGACTACAAAGACGATGACGACAAGGGAAGCGGATCCGCCCTCCACAGCCCGCAG and CGGATCCTATCTCGAGATTACAGCAGGTCCACTCGGCG. The coding sequences of MYC-USP10 and FLAG-USP10 were combined with linearized pQCXIP vector by using Gibson Assembly Master Mix (New England Biolabs, E2611).

pQCXIP-FLAG-USP13 was generated by combining the coding sequence of USP13 with linearized pQCXIP vector as described above. The coding sequence of USP13 was amplified from FLAG-HA-USP13 with primers TAGGCTAGCCTCGAGATGGACTACAAAGACGATGACGACAAGGGAAGCGGATCCCAGCGCCGGGGCGCCCTG and AAGCGGCCGCCCGGGTTAGCTTGGTATCCTGCGGTAAAAGTACATGTAGCCCAGGTCTTTAG. The insertions of USP10 and USP13 were confirmed by DNA sequencing.

### Genome editing using CRISPR-Cas9

USP10-KO H4 cells were generated by CRISPR-Cas9 as previously described (30). The targeting sequences for USP10 (AAATTGTTCAGCGTAAGTAA and GCCTGGGTACTGGCAGTCGA) were cloned separately into pSpCas9 (BB)-2A-GFP plasmid. H4 cells were cotransfected with two plasmids containing the different targeting sequences. After 24 h, GFP-positive cells were sorted on a FACS Aria II Flow Cytometer (BD Biosciences) to a 96-well plate at one cell per well. After 14 days, the cells in each well were trypsinized, genomic DNA was extracted, and cleavage of the target sequence was tested by PCR with primers TAGGATTCTTTGCGTAGTTCATGTT and CCAGGGCTTCTGTGGAGATAC. The KO was confirmed by DNA sequencing and immunoblotting.

### Generation of stably transduced MYC-USP10 rescue cells

Retroviral particles were prepared by transfecting HEK293T cells with pQCXIP-MYC-USP10, pCMV-Gag-Pol (Cell Biolabs, RV-111), and pCMV-VSV-G (Cell Biolabs, RV-110) plasmids. After 48 h, supernatants were collected and centrifuged at 3000 x *g* to remove cell debris. USP10-KO H4 cells were infected with virus supernatants in the presence of 5 μg/ml Polybrene at 37 °C. Four hours after infection, the supernatants were aspirated and replaced with fresh culture medium, and cells were incubated at 37 °C overnight. The stably transduced USP10-KO cells were selected with 1 μg/ml puromycin.

### CRISPR-Cas9 KO screen with ubiquitination library

The ubiquitination screen was as previously described (12). Briefly, a CRISPR-Cas9-KO pooled library targeting major ubiquitination-related genes was transfected into HEK293T cells together with pMD2.G and psPAX. After 48 h, supernatants were collected, and the viral titer was determined. For the ubiquitination screen, 20 million H4-tfLC3B cells were infected with the lentiviral pool at a multiplicity of infection of 0.3. After a 7-day selection with 1 μg/ml puromycin, the initial screen was performed by collecting cells with decreased GFP and mCherry signals by sorting on an FACS Aria II Flow Cytometer. The sorted cells were propagated to 10 million and subjected to the next round of sorting with the same gating until the GFP-mCherry negative population was enriched to >90%.

### Next-generation sequencing

Next-generation sequencing was conducted as previously described (12). Briefly, genomic DNA from 10 million unsorted cells (1,000x coverage of the ubiquitination library) and 10 million sorted cells was extracted using Blood & Cell Culture DNA Midi Kit (QIAGEN, 13343) as per the manufacturer’s instructions. Genomic DNA (40 μg) from each group was used as template DNA for the PCR to amplify the coding region of sgRNAs. PCR reactions were set up using NEBNext High Fidelity PCR Master Mix (New England Biolabs, M0541) with the following primer set: AATGGACTATCATATGCTTACCGTAACTTGAAAGTATTTCG/CAAAAAAGCACCGACTCGGTGCCACTTTTTCAAG. The PCR products were purified with NucleoSpin Gel and PCR Clean-up Kit (MACHEREY-NAGEL, 740609) and used as a template for the second-round PCR to amplify and attach Illumina-compatible multiplexing sequencing adapters and barcodes. The products from the second-round PCR were extracted from the gels and sequenced on a HiSeq 2500 (Illumina) sequencer by the Molecular Genomics Core of NICHD, NIH. The sgRNA sequences, which were 20-bp in length, were then mapped to the reference file of all sgRNAs present in the library. The number of reads of each sgRNA was calculated and analyzed using the MAGeCK algorithm (15).

### Immunofluorescence microscopy

WT and USP10-KO H4 cells were grown on glass coverslips coated with 5 μg/ml fibronectin for 24 h prior to experiments. Cells were incubated with 5 μg/ml puromycin for 2 or 3 h, then washed once with PBS, fixed in 4 % paraformaldehyde (PFA) in PBS for 20 min at room temperature, permeabilized with 0.1 % saponin (Sigma-Aldrich, 47036) for 20 min, and incubated with blocking buffer containing 0.2% BSA (Sigma-Aldrich, A7030). Cells were next stained with anti-SQSTM1 and anti-Ub primary antibodies diluted in 0.2 % BSA for 1 h at 37 °C, followed by staining with Alexa Fluor-conjugated secondary antibodies (Thermo Fisher Scientific) for 30 min at 37 °C. Cells were washed three times with PBS and once with distilled water and mounted with DAPI-Fluoromount-G (Electron Microscopy Sciences, 17984-24). Fluorescence was visualized on a Carl Zeiss LSM780 confocal microscope. Image analysis was performed with ImageJ.

### Immunoblotting

Cells were lysed with 1x LDS (lithium dodecyl sulfate) sample buffer (Thermo Fisher Scientific, NP0007) on an orbital shaker for 20 min at room temperature. The lysates were transferred to 1.5 ml Eppendorf tubes, incubated on a heat blocker at 95 °C for 20 min, and then cleared by centrifugation at 13,000 x *g* for 10 min. The proteins in lysates were separated by SDS-PAGE and transferred to nitrocellulose membranes. The membranes were blocked with 5% nonfat milk, incubated with primary antibodies, horseradish peroxidase (HRP)-conjugated secondary antibodies, and visualized by incubation with chemiluminescent HRP substrate.

### Immunoprecipitation

Cells were lysed on ice for 20 min with lysis buffer (150 mM NaCl, 50 mM Tris-HCl pH 7.4, 5 mM EDTA, 1% Triton X-100, 3% glycerol [Sigma-Aldrich, G6279]) with a protease inhibitor cocktail (Roche, 11697498001). Cell lysates were cleared by centrifugation at 13,000 x *g* for 20 min at 4 °C. The supernatants were incubated with anti-FLAG-conjugated magnetic beads (Thermo Fisher Scientific, A36797) for 4 h at 4 °C. The immunoprecipitates were washed three times with lysis buffer and eluted with 100 μg/ml 3xFLAG peptide (Sigma-Aldrich, F4799) in 50 mM Tris-HCl pH 7.4, 150 mM NaCl. Cell lysates and immunoprecipitates were analyzed by SDS-PAGE and immunoblotting with antibodies indicated in the figures.

### Deubiquitination assay

FLAG-LC3B proteins were purified by immunoprecipitation with anti-FLAG-conjugated beads from lysates of H4 cells transfected with plasmids encoding HA-Ub and FLAG-LC3B. The deubiquitination assay was performed by incubating beads with 5 μg recombinant His6-USP10 or His6-USP13 in 30 μl deubiquitination buffer (50 mM Tris-HCl pH 7.4, 150 mM NaCl, 5 mM MgCl_2_, 10 mM DTT) at 37 °C for 1 h. The deubiquitination reaction mixtures were collected for further analysis. After one wash with deubiquitination buffer, the FLAG-LC3B proteins were eluted with 3xFLAG peptide. Cell lysates, elution products, and deubiquitination reaction mixtures were analyzed by SDS-PAGE and immunoblotting.

### Quantification and statistical analyses

Quantification of immunoblotting experiments was reported as the mean ± SD of the ratio of experimental versus control band intensity from multiple experiments. Control band intensity was arbitrarily set at 1 for the purpose of normalization. Band density values were corrected for loading using β-tubulin or β-actin immunoblots.

All graphs represent data from at least three independent experiments. Statistical comparisons were made using a one-sample *t*-test (when comparing experimental values to a control value defined as 1) or an unpaired Student’s *t*-test (all other comparisons) with Prism 7 software, as indicated in each figure legend. Numerical *p*-values are indicated in each graph; n.s. stands for not significant.

## Data availability

All data are contained within the article.

## Supporting information

This article contains supporting information.

## Conflict of interest

The authors declare that they have no conflicts of interest with the contents of this article.
