# Supplementary material for: The ubiquitin isopeptidase USP10 deubiquitinates LC3B to increase LC3B levels and autophagic activity
Source: J Biol Chem. 2021 Feb 10;296:100405. doi: 10.1016/j.jbc.2021.100405 (PMC7960534; doi:10.1016/j.jbc.2021.100405)
Supplement: Figures S1–S5 [file mmc1.docx]

**Supporting information**

**The ubiquitin isopeptidase USP10 deubiquitinates LC3B to increase LC3B levels**

**and autophagic activity**

Rui Jia^1 *^ and Juan S. Bonifacino^1^

^1^ Neurosciences and Cellular and Structural Biology Division

*Eunice Kennedy Shriver* National Institute of Child Health and Human Development,

National Institutes of Health, Bethesda, Maryland, 20892, USA

^*^ For correspondence: Rui Jia, rui.jia@nih.gov or rui.jia@hotmail.com

Figure S1


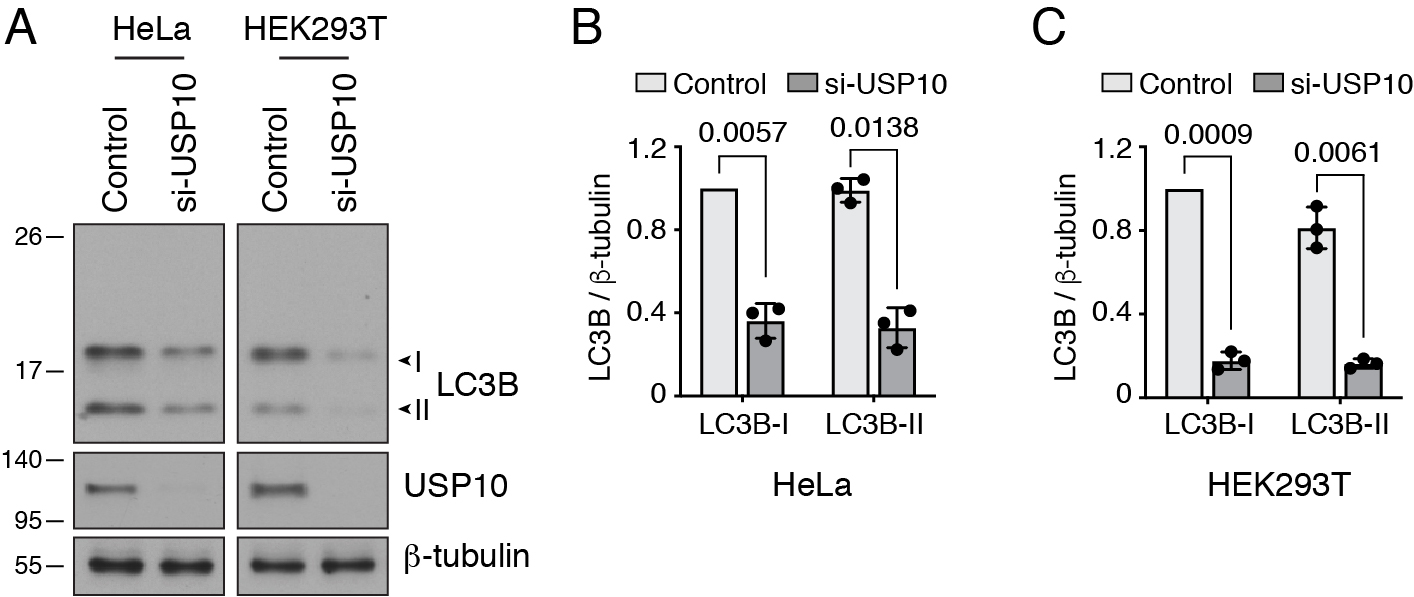


**Figure S1. Reduced levels of LC3B in USP10-KD HeLa and HEK293T cells.**

*A*, HeLa and HEK293T cells were transfected with control or USP10 siRNA SMARTpool. After 48 h, cells were lysed by incubating with 1x LDS sample buffer and analyzed by SDS-PAGE and immunoblotting for the indicated proteins. The positions of molecular mass markers (in kDa) are indicated on the left. *B*,*C*, Quantification of the ratio of LC3B-I and LC3B-II to β-tubulin in HeLa (*B*) and HEK293T (*C*) cells. The LC3B-I to β-tubulin ratio for control siRNA transfection was arbitrarily set at 1. Bars represent the mean ± SD of values from three independent experiments such as that shown in panel *A*. Individual values from each experiment are represented by dots. The indicated *p*-values for LC3B-I were calculated using a one-sample *t* test; the *p*-values for LC3B-II were calculated using a unpaired Student’s *t* test. Notice that USP10 silencing reduced the levels of both LC3B-I and LC3B-II by ~2.5-3-fold in HeLa cells and ~5-5.5-fold in HEK293T cells.

Figure S2


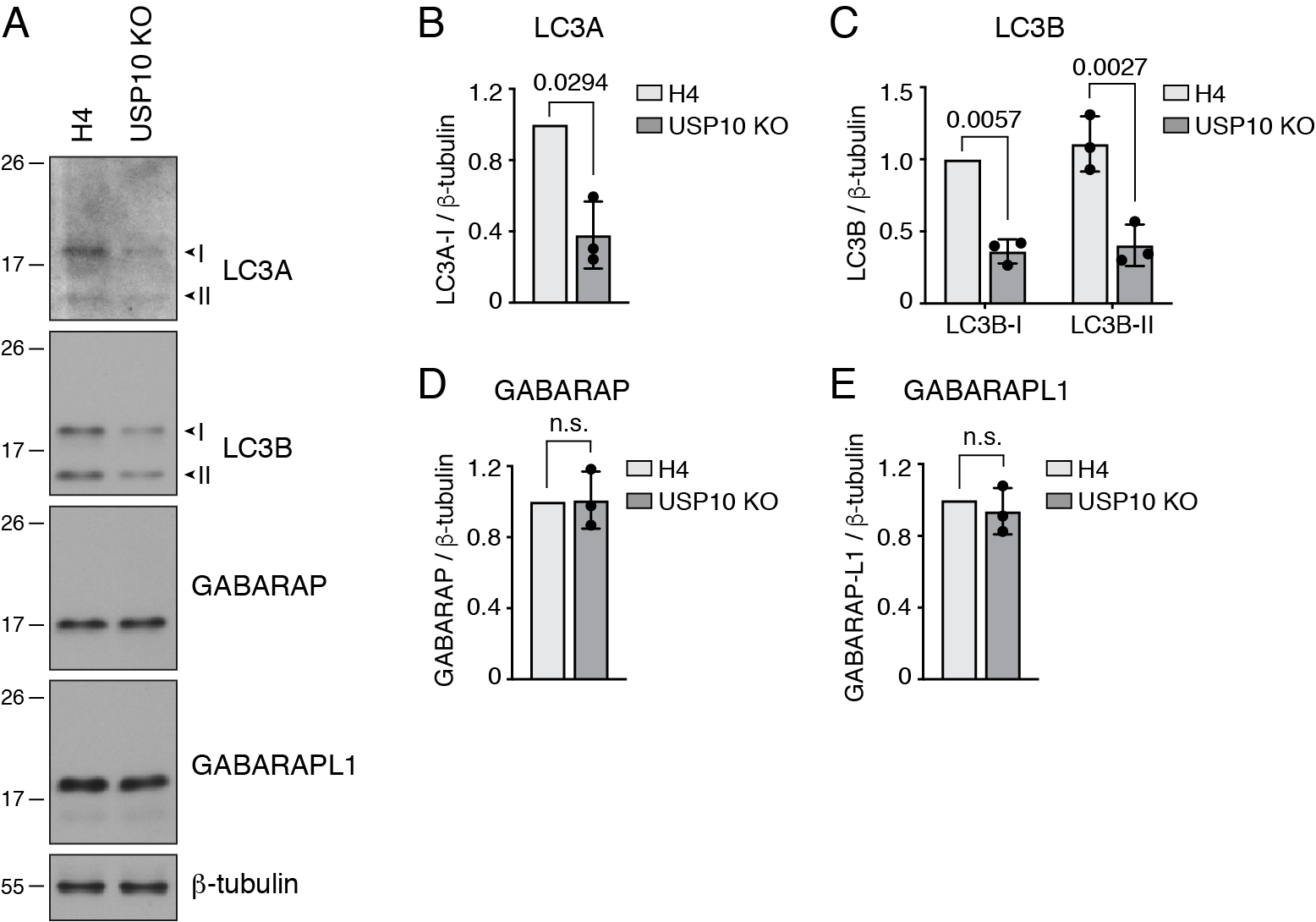


**Figure S2. Reduced levels of LC3A and LC3B in USP10-KO H4 cells.**

*A*, WT and USP10-KO H4 cells were lysed in 1xLDS sample buffer and analyzed by SDS-PAGE and immunoblotting with antibodies to LC3A, LC3B, GABARAP, GABARAPL1 and β-tubulin. The positions of molecular mass markers (in kDa) are indicated on the left. *Β*-*Ε*, Quantification of the ratio of LC3A (*B*), LC3B (*C*), GABARAP (*D*) and GABARAPL1 (*E*) to β-tubulin. The ratio of LC3A-I (*B*), LC3B-I (*C*), GABARAP (*D*) and GABARAPL1 (*E*) to β-tubulin for WT H4 cells was arbitrarily set at 1. LC3A-II could not be accurately quantified because of background interference. Bars represent the mean ± SD of values from three independent experiments such as that shown in panel *A*. Individual values from each experiment are represented by dots. The indicated *p*-values were calculated using a one-sample *t* test, except for LC3B-II in panel *C*, for which an unpaired Student’s *t* test was used. Notice that LC3A and LC3B levels were reduced by ~2.5 fold in the USP10-KO cells. In contrast, the levels of GABARAP and GABARAPL1 did not change in USP10-KO cells. LC3C and GABARAPL2 were not tested because of lack of suitable antibodies.

Figure S3


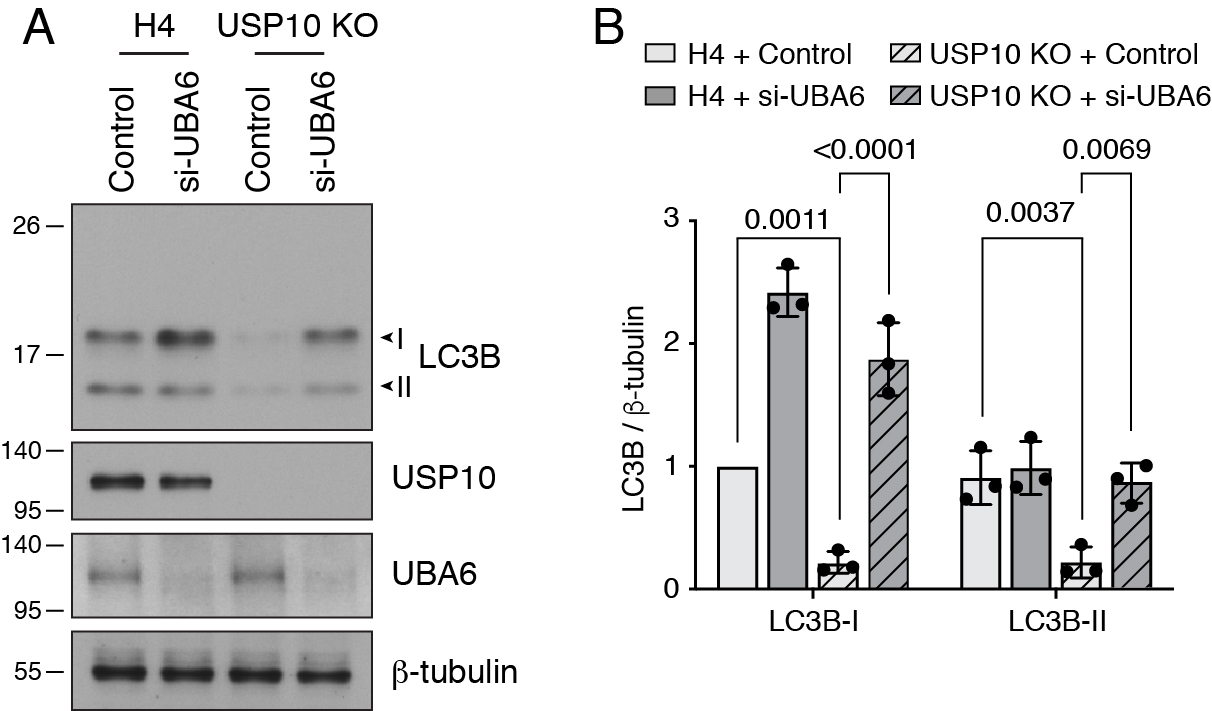


**Figure S3. UBA6 KD increases LC3B levels in USP10-KO cells.**

*A*, WT and USP10-KO H4 cells were transfected with control or UBA6 siRNA. After 48 h, cells were analyzed by SDS-PAGE and immunoblotting for the indicated proteins. The positions of molecular mass markers (in kDa) are indicated on the left. *B*, Quantification of the ratio of LC3B to β-tubulin. The LC3B-I to β-tubulin ratio for the control siRNA transfection in WT H4 cells was arbitrarily set at 1. Bars represent the mean ± SD of values from three independent experiments such as that shown in panel *A*. Individual values from each experiment are represented by dots. The *p*-value relative to LC3B-I in H4 control was calculated using a one-sample *t* test; other *p*-values were calculated using an unpaired Student’s *t* test. Notice that, in USP10-KO cells, silencing UBA6 elevated the levels of both LC3B-I and LC3B-II.

Figure S4


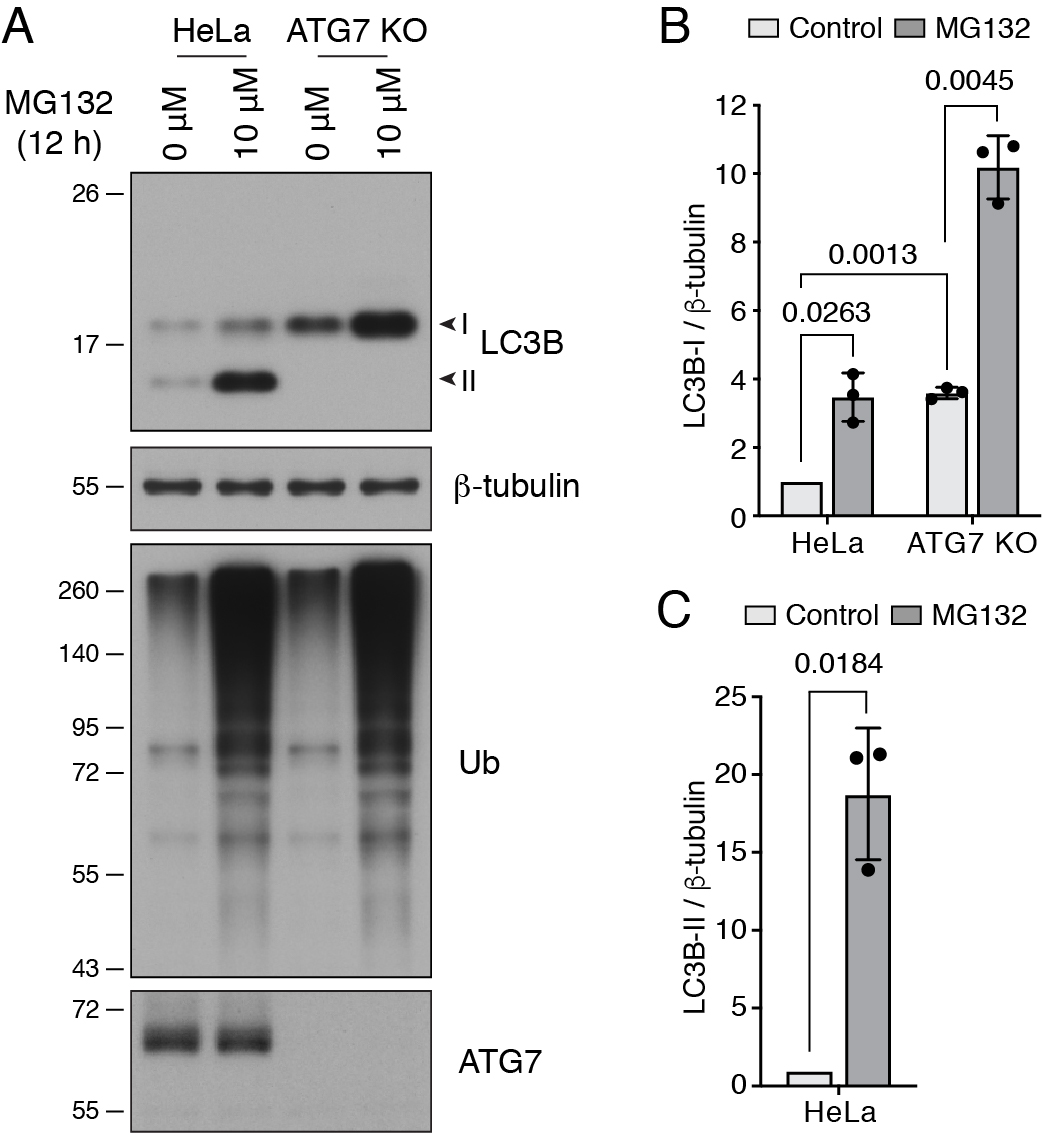


**Figure S4. MG132 increases LC3B-I levels in ATG7-KO cells.**

*A*, WT and ATG7-KO HeLa cells were incubated with vehicle or 10 μM MG132 for 12 h, and then analyzed by SDS-PAGE and immunoblotting. The positions of molecular mass markers (in kDa) are indicated on the left. *B,C*, Quantification of the ratio of LC3B-I (*B*) and LC3B-II (*C*) to β-tubulin. The ratio of LC3B-I (*B*) or LC3B-II (*C*) to β-tubulin for WT HeLa cells without MG132 was arbitrarily set at 1. Bars represent the mean ± SD of values from three independent experiments such as that shown in panel *A*. Individual values from each experiment are represented by dots. The indicated *p*-values relative to the HeLa control were calculated using a one-sample *t* test; the *p*-value for the difference between control and MG132 in ATG7-KO cells was calculated using an unpaired Student’s *t* test. Notice that MG132 treatment elevated the levels of LC3B-I by ~3 fold in both WT and ATG7-KO HeLa cells, while it elevated the levels of LC3B-II by 18-fold in WT HeLa cells. Depletion of ATG7 abolished the conversion of LC3B-I to LC3B-II, preventing its autophagic degradation. The increased levels of LC3B by MG132 in ATG7-KO cells were therefore due to inhibition of proteasomal degradation and not autophagic degradation.

Figure S5


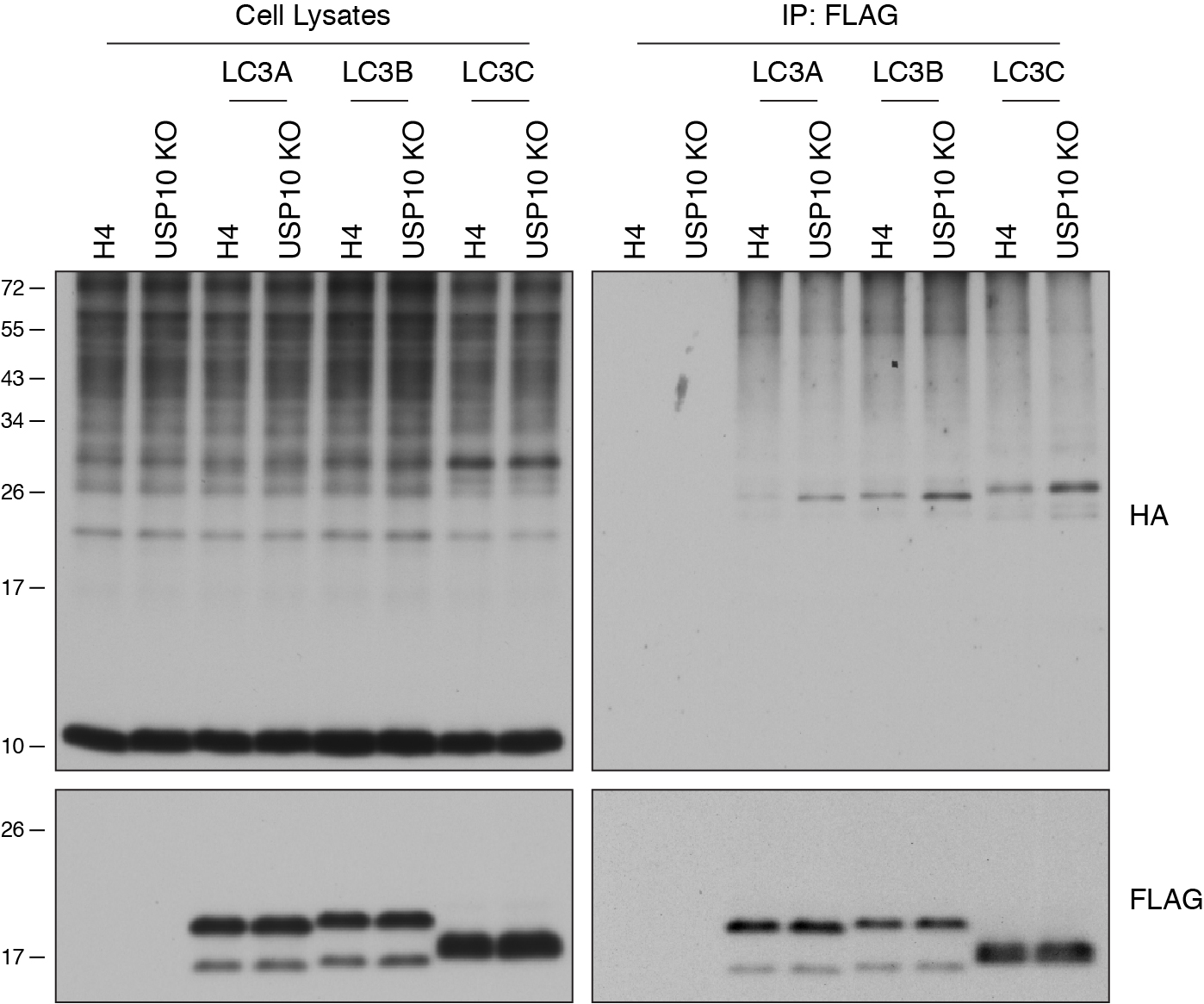


**Figure S5. Increased ubiquitination of LC3 subfamily proteins in USP10-KO cells.**

H4 cells were transfected with plasmids encoding FLAG-LC3A, FLAG-LC3B or FLAG-LC3C together with HA-Ub. After 24 h, cells were incubated with 10 μM MG132 for 24 h. Cells were extracted with immunoprecipitation lysis buffer and cell extracts immunoprecipitated with antibody to the FLAG epitope. Cell lysates and immunoprecipitates were analyzed by SDS-PAGE and immunoblotting with antibodies to the FLAG and HA epitopes. The positions of molecular mass markers (in kDa) are indicated on the left. Notice that LC3B, LC3A and LC3C were monoubiquitinated, in agreement with our previous report (ref. 12). Ubiquitination of LC3A, LC3B and LC3C was increased in USP10-KO cells, indicating that USP10 deubiquitinates all three members of LC3 family proteins.

Figure S6


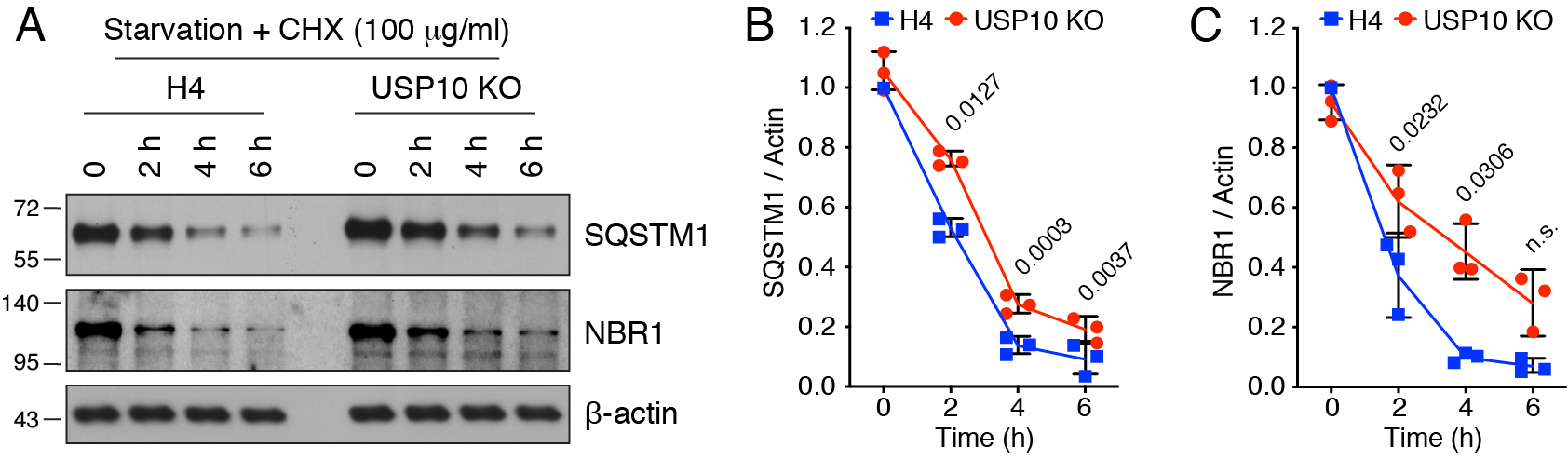


**Figure S6. Reduced rate of degradation of autophagy receptors in USP10-KO cells under starvation conditions.**

*A*, WT and USP10-KO H4 cells were incubated with starvation medium containing 100 μg/ml cycloheximide (CHX) for 0, 2, 4 or 6 h, and analyzed by SDS-PAGE and immunoblotting. The positions of molecular mass markers (in kDa) are indicated on the left. *B*,*C*, Quantification of the ratio of SQSTM1 (*B*) and NBR1 (*C*) to β-actin at different time points. Values for WT H4 cells at time 0 were arbitrarily set to 1. Error bars represent the mean ± SD from three independent experiments such as that shown in panel *A*. Individual values from each experiment are represented by dots. The indicated *p*-values relative to WT H4 cells were calculated using an unpaired Student’s *t* test. Notice that the degradation of autophagy receptors SQSTM1 and NBR1 was attenuated in USP10-KO cells under conditions of starvation and protein synthesis inhibition.
